# Environmental hygiene strategies to combat antimicrobial resistance in healthcare settings

**DOI:** 10.1017/ash.2025.33

**Published:** 2025-03-03

**Authors:** Mary Morgan Lee, Caroline A. O’Neil, Lucy Vogt, Jennie H. Kwon

**Affiliations:** Division of Infectious Diseases, John T. Milliken Department of Internal Medicine, Washington University School of Medicine, St Louis, MO, USA

## Abstract

In this manuscript, we highlight current literature on environmental hygiene techniques to combat reservoirs of antibiotic resistant organisms in the healthcare environment. We discuss several topics for each strategy, including mechanism of action, assessment of effectiveness based on studies, cost, and real-world translatability. The techniques and topics summarized here are not inclusive of all available environmental hygiene techniques but highlight some of the more popular and investigated strategies. We focus on the following: Ultraviolet radiation, hydrogen peroxide vapor, copper-coated surfaces, phages, interventions involving sinks, and educational initiatives.

## Introduction

Antimicrobial resistance is a global health threat, associated with almost 5 million deaths worldwide in 2019.^1^ A 2019 CDC report noted 197,400 cases of extended-spectrum beta-lactamase (ESBL)-producing Enterobacteriaceae in hospitalized patients, associated with 9,100 deaths.^2^ Other organisms of concern include multi-drug resistant (MDR) *Pseudomonas aeruginosa*, methicillin-resistant *Staphylococcus aureus* (MRSA), carbapenem-resistant Enterobacteriaceae, and carbapenem-resistant *Acinetobacter*.^2^ Emerging threats, including *Candida auris,* highlight the importance of infection prevention in healthcare settings.^2^

There is growing attention on the built environment as reservoir for antibiotic resistant organism(ARO) transmission, resulting in infections and outbreaks. In 2016, a cluster of MDR *Sphingomonas koreensis* infections was identified at the National Institutes of Health Clinical Center, with the source traced to sinks in patient rooms.^3^ An outbreak of *Klebsiella pneumoniae* carbapenemase-producing *K. pneumoniae* infected 17 patients, killing 11, in an intensive care unit (ICU) at the same center in 2011, with the offending strain linked to sink drains and isolated from a ventilator after decontamination.^4^ Patient bed rails were linked to an outbreak of *A. baumanii* in an ICU in Argentina in 1996.^5^ Each occurrence like this calls for efforts to make the healthcare environment a safer place for patients.

In this manuscript, we highlight current literature on a sample of environmental hygiene techniques to combat reservoirs of AROs in the healthcare environment. To identify strategies, we performed a nonsystematic search of PubMed and references of previously published studies. The techniques and topics summarized here are not inclusive of all available environmental hygiene strategies but highlight those more common and/or investigated in literature. We will focus on the following: Ultraviolet (UV) radiation, hydrogen peroxide vapor, copper-coated surfaces, phages, interventions involving sinks, and educational initiatives, as summarized in Table 1.

**Table 1.** Profiled environmental hygiene strategies

Strategy	Time for disinfection	Organisms	Cost	Advantages	Limitations
Ultraviolet (UV) Radiation	Automated disinfection in 1–2 h^21^	Broad spectrum antimicrobial activity	Relatively high and variable, dependent on scale	No-touch	Requires precleaning and empty room; efficacy dependent on contact, surface type, etc.
Hydrogen Peroxide Vapor (HPV)	Automated disinfection in 3–4 h^10,32^	Broad spectrum antimicrobial including sporicidal activity	Relatively high and variable, dependent on scale	No-touch	Requires precleaning, empty room, and time for contact with surfaces and conversion to safe particles
Copper-coated surfaces	Continual disinfection	Broad spectrum antimicrobial activity	Variable, dependent on scale	Limited maintenance/ ongoing cost	Efficacy may be affected by anti-corrosion treatment or cleaning products
Phages	Unknown, potentially hours	Broad spectrum antimicrobial activity *in vitro* including against biofilms	Unknown	Genetic engineering could expand implementation	No real-world studies; potential unknown effects
Sinks: strategies ranging from improving disinfection techniques to removing sinks entirely	N/A	N/A	Variable depending on strategy	Could result in long-lasting behavioral/structural change	May require significant alterations in daily practices or structures; Potential high-cost/high-reward interventions
Educational Initiatives	N/A	N/A	Relatively low; dependent on strategy	Collaborative engagement with environmental service workers and other staff	Dependent on staff uptake

## Ultraviolet (UV) radiation

UV disinfection using the Ultraviolet-C (UVC) spectrum has been recognized as a technique for environmental disinfection for many years. It is thought that introducing photons can cause genomic damage to microorganisms, resulting in inactivation.^6–9^ Inactivation rates depend on the microbe’s individual susceptibility to specific UV wavelengths, the material and structure of the target surface, and ambient conditions, such as precleaning prior to disinfection.^6,10^ UVC radiation has been shown *in vitro* to kill MDR organisms (MDROs) known to cause hospital acquired infections (HAIs), including *P. aeruginosa*, *Acinetobacter baumanii*, *Enterococcus faecalis*, *Escherichia coli*, *S. aureus*, and *Clostridioides difficile*.^11,12^

Multiple studies have investigated the role of UVC disinfection in the healthcare setting, with variability in study design and results. A 2018 randomized controlled trial published in the *Lancet* suggests the addition of UVC disinfection to standard cleaning practices may reduce HAI rates.^13^ Authors compared four terminal disinfection strategies for rooms that had been occupied by patients infected with *C. difficile* and other MDROs, including MRSA, vancomycin-resistant *Enterococci* (VRE), and MDR *Acinetobacter*. They reported a significant decrease in incidence of the target organisms among patients who were subsequently admitted to rooms that underwent standard terminal disinfection (with quaternary ammonium) + UVC disinfection versus patients admitted to rooms that only underwent standard terminal disinfection (95% CI 0·50–0·98; *P* = 0·036).^13^ There was no difference in incident cases of C. *difficile* among patients admitted to rooms that had terminal disinfection using bleach vs. bleach + UVC radiation. However, a secondary analysis evaluating *hospital-wide* incidence of the target organisms showed a significant decrease in the overall incidence of *C. difficile* and VRE when UVC radiation was added to standard disinfection practice. This suggests the addition of UVC may provide benefit, even to patients who are not exposed to rooms that receive UVC disinfection. Median room cleaning time was about four minutes longer in the groups that included UVC disinfection. This group published a separate article on implementation, highlighting several strategies to overcome logistical barriers, including establishing safety as a priority, improving communication, ensuring resource availability, and providing feedback.^14^

Two studies in California similarly found that addition of UVC to standard disinfection was associated with a statistically significant facility-wide reduction of HAIs.^15,16^ One of these demonstrated HAI rates from *A. baumanii*, *C. difficile*, and *K. pneumoniae* were significantly decreased after UVC disinfection was added to standard terminal disinfection practices.^16^ The second study reported addition of UVC disinfection led to a substantial cost savings, without adversely affecting admission processes; however, their cost calculations did not account for the cost of the intervention itself.^15^

Another study performed on bone marrow transplant and oncology units at the University of Pennsylvania demonstrated a 25% decrease in incidence of *C. difficile* infection (CDI) when UVC disinfection was performed following standard terminal bleach cleaning in 21.6% of discharges from the study units.^17^ The authors of this 2013 study estimated the annual cost for this intervention as $294,342 for the first year and $194,250 for the second year, with an observed 53 fewer cases of CDI, resulting in an estimated annual cost savings of $348,528–$1,537,000.^17^

In contrast, two other studies, one performed in cancer and solid organ transplant units and the other in mixed wards and an ICU, showed no difference in rates of *C. difficile* or VRE infection when UVC was added to standard terminal cleaning practices.^18,19^ One of these studies cited low initial CDI rates and high compliance with manual cleaning as possible factors leading to lack of difference.^18^ In a meta-analysis of 13 papers, including several mentioned above, a subgroup analysis demonstrated a statistically significant reduction in CDI rates in studies with high baseline CDI rates (>1.5 per 1,000 patient days) but not for those with low baseline rates.^20^ Another subgroup analysis showed no difference in CDI rates for controlled trials while a significant reduction in CDI rates was found in non-controlled trials. This argues that baseline infection prevention practices and HAI rates, as well as study methodology (including the potential for more confounders in non-controlled trials), may help explain the mixed results for UVC disinfection on HAI rates.

Safety is important when using UV devices to prevent damaging exposure to UV radiation.^6^ Disinfection must take place in an empty room, generally limiting the use of these devices to single-occupancy rooms^17^. The total cost of UVC interventions depends on the size of the space to be disinfected, as well as installation and operation costs. In-center validation may be needed to confirm disinfection, which could be affected by room design, positioning of the UVC device within the room, type of surface material, and level of soiling.^6,10,21^ As mentioned, these factors could confound the results seen at different study sites.

## Hydrogen peroxide vapor (HPV)

Vaporized hydrogen peroxide is produced by vaporization of liquid nitrogen peroxide, creating a mixture of HPV and water vapor. It has broad-spectrum antimicrobial activity, including sporicidal activity.^22^ HPV decomposes to water and oxygen, so it is considered relatively safe and residue-free.^22–24^

HPV disinfection has been shown in studies to eradicate MDR organisms from hospital surfaces more effectively than standard cleaning alone. In one study, 10% of surfaces remained contaminated with gentamicin-resistant Gram-negative rods (GNR) after standard cleaning, compared to 0% after subsequent HPV disinfection.^24^ Similar effectiveness was observed for MRSA and VRE.^24^ Another study found that 26% of patient rooms remained contaminated with MRSA and MDR *A. baumannii* after four rounds of standard cleaning. When HPV treatment was added to just one round of standard cleaning, the rate of persistent contamination decreased to 4.5%.^25^ Another study performed in an ICU found that standard terminal cleaning decreased environmental bacterial load but not MDRO load; whereas the addition of HPV was associated with a significant reduction in MDRO contamination.^26^

In a prospective cohort study performed at a >900 bed tertiary care center, HPV disinfection was added to standard terminal cleaning in rooms that had been occupied by patients infected or colonized with MDROs.^27^ Compared to rooms that received standard cleaning, the proportion of rooms contaminated with MRDOs in the HPV cohort was significantly reduced, and patients who were subsequently admitted to rooms that had been cleaned with HPV were 64% less likely to acquire MDROs.^27^

In contrast, a meta-analysis of 7 clinical HPV studies (all before-after studies except for the prospective cohort study mentioned above) found no significant reduction in CDI or MRSA infection rates.^20^ Alternative intervention compliance (hand hygiene, etc.) was only reported in half of the studies. This highlights the need for more controlled studies to investigate HPV disinfection to reduce HAIs in the absence of confounding variables. However, there is practical evidence of HPV being used in outbreak settings to combat environmental reservoirs contributing to HAIs.^28–30^ A UK hospital used HPV to clean their entire ICU after years of recurrent MDR-GNR infections. Following HPV disinfection, no GNRs were cultured from any of the sampled environmental sites, and there were no cases of *Acinetobacter* infection for four months.^29^ A neonatal ICU used a similar method of HPV disinfection to eradicate *Serratia marcescens* following an outbreak among neonates.^28^

Limitations of disinfection using HPV include the need for significant contact time with surfaces, as well as sufficient time for the hydrogen peroxide to degrade into oxygen and water vapor. Disinfection must be performed in an empty room, and the treated surfaces must be relatively smooth, impervious to moisture, and shaped so that the entire surface is exposed to the HPV.^23,31^ This can add anywhere from 1.5–4 hours of additional cleaning time and room downtime.^26,32^ Additional costs include the need for specialized personnel training, potential alterations to facility duct systems, and purchase and maintenance of the HPV generators.^32^

## Copper

Copper-coated surfaces have been shown to exhibit lower bacterial burden compared to control surfaces.^33,34^ Although small amounts of copper are needed for bacterial growth, when present in excess, ionic copper causes rupture of bacterial cell membranes, osmotic imbalance, oxidative damage, and DNA deterioration.^35,36^ Copper alloys have shown *in vitro* to be toxic to some fungi and many Gram-positive and negative bacteria, including *Enterobacteriaceae*, *Pseudomonas aeruginosa*, *Acinetobacter* species, *Listeria*, MRSA, and VRE.^36–44^ Some copper alloys hold Environmental Protection Agency public health registrations for *S. aureus*, *Enterobacter aerogenes*, *E. coli* 0157:H7, *P. aeruginosa*, MRSA, and VRE, having demonstrated the ability to kill these organisms under conditions designed to simulate the hospital environment.^35,45^

In one multicenter randomized controlled trial (RCT), application of a copper coating to several high-contact objects/surfaces in medical ICUs (<10% of total surface area in the room) resulted in an 83% reduction in surface general bacterial burden (measured in colony-forming units/cm^2^) and a 58% reduction in HAIs among patients admitted to the intervention rooms over a period of 21 months.^45^ A 2022 study in Brazil isolated *Acinetobacter* spp. less frequently from copper-coated surfaces compared to control surfaces, and adding copper coating to bed rails resulted in a significant decrease in overall bacterial burden.^37^

A study published in 2015 estimated a cost of $52,000 for adding copper-coated materials to 6 surfaces in 8 ICU rooms.^45^ Following the switch to copper-coated surfaces, the authors noted a decrease in HAIs leading to potential costs savings and estimated that it would take less than two months to recover the costs associated with installation of the copper-coated surfaces.^45^

Copper-coated surfaces appear to retain their antimicrobial properties over time.^35,46,47^ An *in vivo* study showed that copper-coated medical equipment held up well after exposure to standard cleaning agents;^47^ however, several *in vitro* studies suggest that application of anti-corrosion treatment or cleaning products to copper-coated surfaces may lead to residue build-up and decreased antimicrobial activity.^48,49^

## Phages

Bacteriophages (or phages) are naturally occurring non-living segments of DNA or RNA enclosed in a protein capsid that can integrate into bacterial cell genomes and cause lysis by disrupting replication.^50^ They can be specific to certain bacteria or capable of infecting many different bacteria.^51^ Phages were frequently used to treat lethal bacterial infections during the pre-antibiotic era but were largely abandoned following the discovery of penicillin.^50^ Phage therapy has been revisited in recent years as a potential treatment for MDR bacterial infections. There are currently no FDA-approved phage treatments for human use in the United States or Europe; however, phages are used for infection prevention in other industries, such as food and agriculture.^52^

Although bacteriophages are not yet approved for human use, they are being explored as an environmental hygiene strategy, particularly for the eradication of biofilms and persistent reservoirs in the built environment. In one *in vitro* study, phages were found to decrease the burden of *P. aeruginosa* on plastic surfaces more effectively than standard chemical disinfectants, when tested on spot inoculation and wet biofilms.^52^ The log reduction in bacterial counts increased further when the two methods were combined. Notably, this effect was only seen when chemical disinfection was applied after phage treatment.^52^ When chemical disinfection was applied before phage treatment, the log-reduction in bacteria was lower than for phage therapy alone, suggesting phages may be inactivated by chemical disinfectants.^52^

Dry biofilms are more difficult to eradicate because they can remain dormant on surfaces and reactivate later when exposed to nutrients or moisture, sometimes in the form of cleaning materials.^53^ In the previously described study, phages were not able to remove dry biofilms but prevented regrowth of *P. aeruginosa* from dry biofilms for 8 hours after treatment.^52^ Additional studies have demonstrated *in vitro* effectiveness of phages against biofilms created by various strains of *E. coli*, *Pantoea agglomerans*, *Serratia marcescens*, *Staphylococcus epidermidis*, *and Staphylococcus capitis*.^54–60^ Despite promising *in vitro* studies, there is a need for real-world research to understand the role of bacteriophages as environmental hygiene tools.

Bacteriophages can be genetically engineered to allow for standardization and expedition in development and distribution.^50^ Phages contain genetic material encoding enzymes that aid in bacterial cell destruction, and there is research investigating how to engineer phage-derived lytic proteins for efficacy independent of the phage itself.^61^ This could bypass the potential for bacteria to develop phage resistance, which is known to occur and represents a limitation for use of phages, both for environmental disinfection and clinical treatment.^62^ At least one study, however, did not observe emergent phage resistance in *S. capitis* when targeted by single phages or phage cocktails.^60^

Since bacteriophages can alter bacterial cell genomes, they may cause recovery of antimicrobial susceptibility in MDR organisms. One study demonstrated development of phage resistance in several strains of *P. aeruginosa* caused changes in efflux pump mechanisms, which resulted in increased susceptibility to several antibiotics.^63^ However, bacteriophages also have the potential to make deleterious genetic alterations. Lysogenic phages integrate genetic material but do not kill the bacteria. When a portion of the bacterial population survives, these phage-encoded genes are propagated forward in future generations.^50^ Prophage genes can encode bacterial virulence factors (ie Shiga toxin, botulinum toxin), in addition to antibiotic resistance genes (ie beta lactamases).^64,65^

Limitations for the use of phages in the healthcare environment include the potential for unintended consequences, as described above, as well as time and environmental parameters. Most of the mentioned *in vitro* studies incubated phages with bacteria for several hours at a specific temperature and pH. Given the absence of real-world data, it is difficult to know whether phages would effectively kill bacteria in the typical healthcare environment. Additionally, the extended time required for phage application could result in delays in room turnover and patient care.

## Sinks and sink drains

Hospital sinks are known to serve as reservoirs for MDR gram negative bacteria, particularly in ICUs, and have been implicated in outbreaks related to carbapenem-resistant and ESBL-producing organisms, including *Pseudomonas* and *Klebsiella* spp.^66–68^ Sinks are a necessary component of the hospital-built environment, so addressing sinks as a source of infection in the hospital is key.

Several studies have assessed the effectiveness of various sink disinfection strategies in reducing AROs in and around hospital sinks. One study in a medical ICU compared disinfectants for cleaning sinks in patient rooms.^69^ One day after the intervention was performed, the authors found a significant decrease in bacterial burden in sinks that were treated with hydrogen peroxide, compared to sinks treated with bleach or standard cleaning protocols.^69^ However, after seven days, bacterial burden in all of the sinks had returned to pre-treatment levels.^69^ An RCT in Canada compared a combination of chemical, mechanical, and heat cleaning added to their standard sink cleaning protocol using hydrogen peroxide sporicidal gel.^70^ The intervention, which combined mechanical scrubbing, sodium hydroxide, enzymatic solution, hydrogen peroxide, and a steamer, required >1 hour of additional cleaning time compared to standard practice.^70^ The authors found that drains treated with the intervention were more likely to have no detectable carbapenemase gene in the drain for seven days following the intervention.^70^

Another option is to remove sinks entirely from patient rooms, creating “waterless units.” One institution tested this method in their medical and surgical ICUs over a six-year period.^71^ They removed all sinks in patient rooms, leaving only two sinks in a central workroom. They also implemented water-safe strategies on the remaining sinks, including deep drain cleaning, applying monthly antibacterial filters, and replacing siphons and tap aerators every three months. These changes were associated with a significant decrease in the incidence of ICU-acquired MDR gram negative bacteria, including *K. pneumoniae* and *P. aeruginosa*.^71^ Limitations to this method include significant cost, labor, time, and concerns about hand hygiene and cleaning compliance if sinks aren’t easily accessible.

## Educational initiatives

Education is a cornerstone in improving healthcare environmental hygiene, and various strategies have been assessed for their ability to reduce or eradicate reservoirs of AROs and reduce HAIs. One institution in Taiwan conducted a prospective trial implementing a human factors engineering-focused strategy to improve cleaning of high-touch surfaces in their medical/surgical wards and ICUs.^72^ Their intervention included meetings between hospital administrators, infection control leaders, and environmental service worker (ESW) supervisors to provide education about strengthened cleaning techniques focused on simple ergonomic workflows and a checklist for high-touch surfaces. They assessed adequacy of cleaning using fluorescent markers and provided feedback to the ESWs. During the intervention, they demonstrated a significant increase in cleaning adequacy, as measured by a terminal cleaning and disinfecting score. They also observed significantly decreased incidence of MDRO carriage; however, there was no difference in MDRO HAIs during the intervention period.^72^

Other institutions have demonstrated improved room cleaning and decreased incidence of CDI after requiring ESWs to complete online education modules, followed by assessments and feedback.^73,74^ One multicenter study employed a 5-module educational program covering topics including hand hygiene, isolation precautions, personal protective equipment (PPE), cleaning protocols, and methods to overcome barriers.^74^ Following the course, ESWs reported feeling more comfortable with PPE and better understanding of the importance of cleaning high-touch surfaces, which significantly increased the frequency of cleaning individual high-touch surfaces.^74^

Other studies have suggested that educational toolkits and staff feedback may improve HAI rates. Two hospitals achieved 100% reduction in CDI rates with education based on CDC toolkits, which focus on ESWs’ roles as advocates for patient safety and highlight the importance of terminal cleaning and cleaning high-touch surfaces.^75^ Another hospital provided education to ESWs focused on repeated bucket immersion during cleaning and feedback regarding cleaning efficacy using black-light markers.^76^ The intervention was associated with a significant reduction in VRE and MRSA acquisition in their ICUs.^76^ In another study, housekeeping education and weekly meetings between housekeeping, ICU staff, and infection prevention leadership were part of a successful aggressive enhanced decontamination program to end a VRE outbreak in a burn ICU.^77^

The cost of educational interventions is difficult to quantify, and the time required will vary depending on the material and persons involved. However, some interventions may be implemented without the introduction of new expensive tools and techniques, which suggests they could be cost-effective. Another benefit to these interventions is the inclusion of ESWs in initiatives that emphasize the importance of their role in patient safety and infection prevention.

## Summary

The healthcare environment can be a fixed reservoir for MDROs that cause clinically significant infections, which can be difficult or impossible to treat, highlighting the importance of prevention. Environmental hygiene is necessary to combat reservoirs of resistant bacteria in the healthcare environment and provide safe spaces for patients. This article highlights some of the more popular and widely investigated strategies. Due to space constraints, it does not cover all environmental hygiene strategies available or in development. Another limitation is that this was not a systematic literature review, so it does not encompass all available articles addressing these strategies. The inherent bias for publication of positive studies should also be mentioned. Many of the studies highlighted here were quasi-experimental with positive results; however, in at least one instance, meta-analyses of these studies did not reach significance. We are in need of more controlled studies to evaluate many of these strategies, with better accounting for confounding variables. The strategies summarized here should be considered on an individual institution basis, taking into account baseline infection rates and the success of current hygiene strategies, as well as cost and safety analyses.
